# Healthy Diet for Healthy Aging

**DOI:** 10.3390/nu13124310

**Published:** 2021-11-29

**Authors:** Suey S. Y. Yeung, Michelle Kwan, Jean Woo

**Affiliations:** 1 Department of Medicine and Therapeutics, Faculty of Medicine, The Chinese University of Hong Kong, Hong Kong, China; sueyyeung@cuhk.edu.hk; 2 Jockey Club Institute of Ageing, The Chinese University of Hong Kong, Hong Kong, China; michellekwan@cuhk.edu.hk; 3 Centre for Nutritional Studies, Faculty of Medicine, The Chinese University of Hong Kong, Hong Kong, China

**Keywords:** diet, healthy aging, healthspan, intrinsic capacity, nutrition

## Abstract

Extending healthspan is a major public health challenge. Diet is one of the modifiable factors for preventing age-related diseases and preserving overall good health status during aging. Optimizing individuals’ intrinsic capacity, including domains in cognition, psychological, sensory function, vitality, and locomotion, has been proposed as a model of healthy aging by the World Health Organization. To better understand the relationships between a healthy diet and healthy aging, this review summarizes the recent epidemiologic and clinical data for dietary patterns that have been shown to play a role in (domains of) healthy aging. Regardless of priori or posteriori dietary patterns, it appears that dietary patterns centered on plant-based foods have a beneficial role in (domains of) healthy aging. Our review identified a knowledge gap in dietary patterns and multidimensional concepts of healthy aging. More epidemiological studies should consider intrinsic capacity as an outcome measure to further our understanding of a healthy diet and multidimensional concepts of healthy aging. When a sufficient number of epidemiological studies is available, evidence can be synthesized and clinical trials can be designed to evaluate a healthy diet as a strategy for healthy aging to further our progress in translating evidence to practice and promoting healthy aging.

## 1. Healthy Aging and Diet as a Key Factor 

With the great improvements in medicine, public health, science, and technology, older adults are now living longer than the previous generations. However, living longer is not necessarily being healthier [1]. Healthy aging is defined as “the process of developing and maintaining the functional ability that enables well-being in older age” [2]. Indeed, the maintenance of health in later life has become a major public health challenge. Nowadays, the focus of gerontology research has shifted from extension of lifespan to extension of healthspan [3]. This direction is aligned with the goal of healthy aging proposed by the World Health Organization (WHO), which suggested a focus on optimizing individuals’ intrinsic capacity as they age [4,5]. This innovative model emphasizes a comprehensive assessment of different domains of intrinsic capacity, which is a combination of the individuals’ physical and mental capacities, including domains in cognition, psychological, sensory function, vitality, and locomotion [6]. It has been suggested that evaluation of older adults in a holistic approach generates better outcomes compared to the traditional disease-based model of care [7]. In view of this, the framework of this review is based on the multidimensional concepts of healthy aging. 

The impact of lifestyle on health status is well established. Dietary habit is one of the main modifiable lifestyle factors for the prevention of age-related diseases and the preservation of overall good health status during aging [8]. Promoting a healthy diet in older adults is vital in helping them to maintain health and functional independence. Studying diet in terms of dietary pattern analysis is more relevant as individuals do not consume single nutrients or food groups [9]. In addition, the synergistic relationship of nutrients rather than a single nutrient could influence physiological and cognitive function [10]. Therefore, reviewing the evidence of dietary patterns rather than individual nutrients or food groups would provide more meaningful recommendations for healthy aging. 

In an attempt to respond to the question of a special issue, “What is healthy diet for healthy people?”, the present review aims to summarize the recent evidence regarding the relationships between healthy diet and healthy aging, with a focus on cognition, psychological function, sensory function, vitality, and locomotion.

## 2. Evidence of Healthy Diet for Healthy Aging

Various dietary patterns have been frequently suggested to play a significant role in health in older age. These dietary patterns are often categorized into a priori approaches and a posteriori approaches [11]. In priori approach, scores or indices of the overall dietary patterns were defined based on foods or diets associated with a specific health outcome or on the specific national or international dietary guidelines for a healthy diet (e.g., the Healthy Eating Index (HEI) [12], the Dietary Approaches to Stop Hypertension (DASH) diet [13], the Mediterranean diet (MedDiet) [14], and Okinawan diet [15] (Table 1)). These priori dietary patterns have been tested against health outcomes such as cardiovascular disease, cancer or Alzheimer’s disease [16]. However, their roles on (domains of) intrinsic capacity are less clear. For the posteriori approach, dietary pattern analyses are driven by the dietary data per se of the population under study, and patterns are derived through data dimensionality reduction techniques. The evidence of different dietary patterns for optimizing (domains of) intrinsic capacity will be discussed.

### 2.1. Cognition

Cognitive function declines with age and can be divided into various domains including processing speed, attention, memory, language, visuospatial abilities, and executive functioning/reasoning [20]. It has been suggested that the neuroprotective effects of diet are mediated by a reduction in vascular and cardiometabolic risk, and by nonvascular mechanisms such as suppression of oxidative stress and inflammation [21]. Among the dietary patterns that have been studied for their roles in cognitive function, the MedDiet has received the most attention [22]. Based on 19 cross-sectional studies that included 19,734 community-dwelling and institutionalized older adults free of disability and dementia, a systematic review showed that high adherence to the MedDiet was associated with better global cognition and memory [23]. In relation to longitudinal analysis, 98,315 community-dwelling older adults from 34 prospective studies with an average follow-up period from 3.0 to 12.6 years were included. Results indicated that non-demented older adults with higher adherence to the MedDiet had a lower decline in global cognitive function compared to those with lower adherence. However, no significant associations between MedDiet adherence and the incidence of mild cognitive impairment (MCI) and dementia were found [23]. Interestingly, the long-term effect of diet on cognitive function has also been demonstrated. Among 2621 individuals from the Coronary Artery Risk Development in Young Adults (CARDIA) cohort, greater adherence to the MedDiet and the CARDIA prior healthy dietary patterns during adulthood was associated with better midlife cognitive performance assessed at years 25 and 30 (mean age 50 and 55 years, respectively) [24]. 

The effectiveness of the MedDiet on cognitive function has been examined in various trials. A systematic review and meta-analysis summarized that there is a lack of convincing evidence for a significant beneficial effect of the MedDiet on cognitive function or brain morphology or function [25]. However, there were significant associations at the individual trial level, in favor of a MedDiet for eight test outcomes related to global cognition, working memory, verbal and visual memory, visuospatial, language, and executive function domains [26,27,28]. The authors suggested that the inconsistent efficacy of a MedDiet across trials compared to observational studies may be explained by short intervention time to detect a significant change, the use of varied MedDiet intervention and methodologies to measure cognitive change [25]. Recently, the NU-AGE randomized controlled trial included 1279 healthy older adults from five European centers. Both intervention (received dietary advice that highly adheres to the nutritional principle of the MedDiet) and control group (followed habitual diet) participants improved in global cognition and in all cognitive domains after 1 year [29]. Furthermore, those with higher adherence to an individually-tailored Mediterranean-like dietary advice experienced considerable improvements in global cognition and episodic memory after 1 year, compared to those with lower adherence to the intervention. In contrast, a 6-month trial in a non-Mediterranean area, the MedLey study, included 137 Australian older adults and found that the MedDiet group did not perform significantly better than the control group (habitual diet) for cognitive test [30]. The findings indicate that the beneficial effects of the MedDiet on cognitive performance may be limited to Mediterranean older adults. This may partly be explained by the challenges in sustaining adherence to red meat restrictions of the MedDiet [31]. In view of this, a recent trial in Australia compared a MedDiet with 2–3 weekly servings of fresh, lean pork and a low-fat control diet [32]. Participants achieved high adherence to the intervention and the 8-week Australian MedDiet led to higher performance in the cognitive domain of processing speed compared with the control diet. 

Fewer studies have investigated the effects of other dietary patterns on cognition. In particular, the effect of adherence to the DASH diet on cognitive health is inconclusive. The Women’s Health Initiative Memory Study (WHIMS) found that the DASH diet was not associated with MCI or probable dementia in older women with or without hypertension during 9.11 years of follow-up [33]. In contrast, greater adherence to long-term DASH diet in over 16,000 older women from the US Nurses’ Health Study was significantly associated with a better average global cognitive function, verbal memory, or telephone interview of cognitive status at older ages. The associations were equivalent to being 1 year younger in age. However, no associations were observed between long-term DASH adherence and cognitive change after 4.1 years [34]. There is increased attention to the recently developed Mediterranean-DASH diet Intervention for Neurodegenerative Delay (MIND) diet, which is a hybrid of the MedDiet and DASH diet and is tailored to protect the brain [35]. In the US Nurses’ Health Study, long-term adherence to the MIND diet was moderately associated with better verbal memory in later life, but there was no association with cognitive decline over 6 years in global cognition, verbal memory, or telephone interview of cognitive status [36]. The protective effects of the MIND diet seem to be geographically generalizable. In the Personality and Total Health (PATH) Through Life cohort, it was found that greater MIND diet adherence was associated with a 53% reduction in the odds of 12-year cognitive impairment among 1220 Australian older adults, but not the MedDiet [37]. Among 2223 older adults without dementia from the Swedish National Study on Aging and Care in Kungsholmen (SNAC-K) cohort, moderate to high adherence to the MIND diet was associated with less cognitive decline over 6 years compared with low adherence to this dietary pattern [38]. Another role of diet on cognitive health is the regulation of chronic inflammation. For this, the Dietary Inflammatory Index (DII) was developed as a means of estimating the inflammatory potential of the overall diet [39]. Evidence of the association between a higher DII score (reflecting a more inflammatory diet) and greater cognitive decline has been shown among 7095 US women over 9.7 years in the WHIMS [40] and 3080 French over 13 years of follow-up in the SU.VI.MAX study [41]. Although observational studies have shown evidence of these dietary patterns on cognitive health, there are limited intervention studies to examine if the DASH, MIND, or anti-inflammatory diets can protect against the rate of cognitive decline over time [22,42]. More trials on these dietary patterns are needed to confirm their neuroprotective effects.

Regarding the associations between posteriori dietary patterns and cognition, the SNAC-K cohort found that moderate and high adherence to the Nordic Prudent Dietary Pattern (NPDP) (emphasizes high intake of non-root vegetables, apples/pears/peaches, pasta/rice, poultry, fish, vegetable oils, tea, and water and light to moderate wine intake; discourages high consumption of root vegetables, refined grains/cereals, butter/margarine, sugar/sweets/pastries, and fruit juice) were associated with less cognitive decline. High adherence to the NPDP was also associated with the lowest risk of Mini-Mental State Examination (MMSE) decline to ≤24 among older adults in Nordic countries [38]. Data of 5083 individuals (mean age of 56 years at baseline) from the Whitehall II cohort showed that a dietary pattern associated with systemic inflammation (characterized by high intake of red meat, processed meat, peas and legumes, and fried food and low intake of whole grains) predicted faster decline in reasoning over 10 years of follow-up [43]. Among 475 Chinese older adults from the National Taiwan University Hospital (NUTH) cohort, a high score in the “traditional” pattern (characterized by fermented food and pickles) protected against the decline of logical memory-recall over 2 years. However, mixed results were reported on the other patterns: the Chinese “vegetable” pattern protected against a decline of logical memory; while a high score in the “vegetable” pattern increased executive function decline. In addition, a high score in the “meat” pattern increased the risk of verbal fluency decline; while a moderate or high score in the “meat” pattern protected against attention decline [44]. 

### 2.2. Psychological

It is estimated that the prevalence rate of depression peaks in older adulthood [45]. Depression is closely related to adverse outcomes such as poor quality of life, frailty, cognitive decline, and increased risk of mortality in older adults [46,47,48]. It has been hypothesized that the protective effects of diet on depression are related to the anti-inflammatory and antioxidative properties, which may relieve the inflammatory cytokine secretion, attenuate the inflammation in the brain, decrease the oxidative stress in specific brain zone, and therefore alleviating depression symptoms [49,50]. Several systematic reviews and meta-analyses have reported the associations between pro-inflammatory diet and increased risk of depression [51,52,53]. From analyses of longitudinal studies, a systematic review and meta-analysis found a robust association between both higher adherence to a MedDiet (relative risk 0.67, 95% confidence intervals (CI) 0.55–0.82) and a lower risk of depression [51]. A similar trend was found for other healthy dietary patterns such as DASH, Healthy Eating Index, andHEIand several other country-specific dietary guidelines [51,54,55]. Recent findings from 709 American older adults (mean age of 80 years) of the Rush Memory and Aging Project (MAP) showed that higher adherence to the DASH and MIND diets were associated with lower rates of depressive symptoms over 6.5 years of follow-up [56]. Furthermore, the Western diet was associated with higher rates of depressive symptoms over time. However, the MedDiet was not associated with depressive symptoms. Similarly, data of 794 older Australian men (mean age of 81 years) from the Concord Health and Ageing in Men Project showed that the Australian Dietary Guideline Index and MedDiet scores had no associations with incident depressive symptoms at 3-year follow-up [57]. Data of 2646 young-old (mean age of 60 years) from the Maastricht Study found that adherence to the MedDiet and DASH diet was not associated with incident depressive symptoms over 7-year follow-up, whereas higher adherence to the Dutch Healthy Diet lowered the risk of incident depressive symptoms [58]. In contrast, among Taiwanese older adults (mean age of 67 years), a traditional dietary pattern characterized by frequent consumption of meat/poultry and eggs with infrequent consumption of fish increased by 60% the risk of depressive symptoms during 8-year follow-up. Both Western and healthy dietary patterns were not associated with subsequent depressive symptoms [59]. For longer follow-up time, a study of 1112 Japanese older adults (mean age of 73 years) found that adherence to Japanese dietary guidelines was not significantly associated with the risk of depression 20 years later [60]. 

Aside from the priori dietary patterns, posteriori dietary patterns have also been reported to regulate mood in older adults. In general, healthy dietary patterns were associated with a lower risk of depression. The Wellbeing, Eating, and Exercise for a Long Life (WELL) cohort among 2142 adults (mean age of 65 years) showed that a healthy dietary pattern (characterized by frequent intake of vegetables, fruits, and fish) was associated with a lower risk of depressive symptoms in Australian women (β −0.201, 95% CI −0.390 to −0.013), whereas no associations were identified in men during 3-year of follow-up [61]. From the cross-sectional analysis of the Tianjin Chronic Low-grade Systemic Inflammation and Health (TCLSIH) Cohort Study, a healthy dietary pattern (vegetables, fruits, and soybean products) was associated with the decreased prevalence of depressive symptoms, whereas both greater adherence to sweets (ice cream, desserts, and fruits) and traditional Tianjin dietary patterns (animal blood, animal offal, sausages, and preserved eggs) were associated with a higher prevalence of depressive symptoms among 2051 Chinese postmenopausal women [62]. In the Invecchiare in Chianti (InCHIANTI) study, investigators used reduced rank regression (RRR), which combines a priori and a posteriori approach, to identify dietary patterns and explored their associations with depressive symptoms over 9 years. The study found that higher adherence to the typical Tuscan dietary pattern (high in vegetables, olive oil, fish, fruits, cereals, and moderate in wine, red meat, and processed meat) was associated with lower depressive symptoms in 1165 Italian older adults (β −1.78, 95% CI −3.17 to −0.38) [63]. Similarly, cross-sectional data from the Hatoyama Cohort Study and Kusatsu Longitudinal Study showed that a RRR-derived dietary pattern as characterized by high intakes of fish, soybean products, potatoes, vegetables, mushrooms, seaweeds, fruits, and green tea and a low intake of rice was associated with lower prevalence of depressive symptoms in community-dwelling older Japanese (odds ratio (OR) 0.53, 95% CI 0.30 to 0.92) [64]. 

Although extensive observational studies showing the association between dietary patterns and psychological wellbeing among older adults are available, intervention trials on this topic are scarce and limited to the MedDiet. It was shown that an 8-week MedDiet supplemented with either dairy foods or fresh and lean pork improved total mood disturbance, depression, or emotional role functioning compared with low-fat control diet among Australian older adults [32,65]. Furthermore, in the PREDIMED (Prevención con Dieta Mediterránea) study, an inverse association with depression was found for older adults with diabetes assigned to a MedDiet supplemented with nuts compared with those assigned to the low-fat control group after at least 3 years of intervention [66].

### 2.3. Sensory Function

Reduction in visual acuity and field of vision, as well as diseases such as age-related macular degeneration (AMD), cataracts, diabetic retinopathy (DR), and glaucoma, become more common with increasing age [67]. Age-related visual impairments may result in a deterioration in posture, gait, and balance, and an increased risk of falls [68,69]. Diet is one of the factors that contribute to disease progression for age-related vision loss [70]. A recent review summarized the association between dietary patterns and incidence and progression of age-related eye diseases [70]. For AMD progression, several population-based studies such as the American Age-related Eye Disease Study (AREDS), the Rotterdam Eye Study, the Atherosclerosis Risk in Communities (ARIC) study, the Eye-Risk Study, and the Coimbra Eye Study showed consensus findings that adherence to a prudent (healthy) dietary pattern [71,72], the MedDiet [73,74,75,76], or the HEI protected against AMD, whereas the Western (unhealthy) dietary pattern can accelerate AMD progression [71,77]. For cataracts, the dietary pattern that has been studied the most was HEI. Although mixed results were found, the Harvard Nurses’ Health Study (NHS), Women’s Health Initiative (WHI) and the Carotenoids in Age-related Eye Disease Study (CAREDS) cohorts have shown that adherence to the HEI was protective for nuclear cataracts [70]. From the PREDIMED trial, no difference in the incidence of cataract surgery (a surrogate for advanced cataract formation) was found in either the MedDiet groups or low-fat control diet group after a median follow-up of 6 years [78]. Furthermore, a relatively few studies have examined the association between dietary patterns and DR. In a post hoc analysis of the PREDIMED trial, a MedDiet supplemented with olive oil had a significant reduction in incident DR comparing with a low-fat control diet in an elderly Mediterranean population with diabetes after a median follow-up of 6 years [79]. To the best of our knowledge, no study has examined the association between dietary patterns and glaucoma.

Hearing impairment is another predominant sensory deficit in older adults. Hearing impairment is associated with social isolation and generates a vicious cycle characterized by a sense of inadequacy, anxiety, depression, cognitive decline, and physical loss [80,81,82]. In a sub-cohort of Nurses’ Health Study II, the Conservation of Hearing Study (CHEARS) including 3135 women (mean age of 59 years) found that higher adherence to DASH, alternate MedDiet, and the alternate HEI 2010 were independently associated with lower risk of hearing loss at 3-year follow-up. In the mid-frequencies, the odds of hearing loss were about 30% lower among those with diets closely adhered to these healthy dietary patterns. In the high frequencies, the odds were 25% lower with the highest DASH score [83]. In contrast, the Rotterdam Study found that there was no association between adherence to the Dutch Dietary Guidelines and hearing thresholds in cross-sectional and longitudinal analyses (follow-up: 4.4 years) of 2906 and 636 older adults respectively [84]. Regarding the association between posteriori dietary patterns and hearing impairment, in the Caerphilly Prospective Study, adherence to a healthy dietary pattern (characterized by high intake of cereals, fruit, high-fiber bread, confectionery, vegetables, natural juices, margarine, milk and cream, and low intake of beer, lard and butter) was inversely associated with hearing loss among 2512 older men at 5-years follow-up (OR 0.83, 95% CI 0.77 to 0.90) [85]. Similarly, a cross-sectional analysis using the UK Biobank resource found that dietary patterns high in fruit and vegetables (OR 0.89, 95% CI 0.83 to 0.96) and low in fat (OR: 1.16, 95% CI 1.08 to 1.24) were independently associated with reduced odds of hearing difficulties, whereas a dietary pattern high in fish, meat, poultry, and eggs was associated with reduced odds of tinnitus (OR 0.90, 95% CI 0.82 to 0.99) and hearing difficulties (OR 0.88, 95% CI 0.82 to 0.95) among 34,576 UK adults aged 40 to 69 years [86]. 

### 2.4. Vitality

The term vitality was used by the WHO to describe the physiological factors that contribute to an individual’s intrinsic capacity. These factors may include energy metabolism, hormonal function, and cardiorespiratory function [6]. To maintain the proper functioning of the body, dietary intake is metabolized to produce the required amount of energy for the maintenance of an optimal homeostatic level. One of the key reasons for decreased vitality in older age is malnutrition [87]. As part of the Hellenic Longitudinal Investigation of Aging and Diet Study, a cross-sectional analysis of 1831 Greek older adults showed that higher adherence to the MedDiet was associated with a lower risk of malnutrition assessed with the Determine Your Nutrition Health checklist [88]. Another cross-sectional analysis among 3694 Chinese community-dwelling older adults showed that better diet quality as assessed with the Diet Quality Index-International (DQI-I) was associated with a lower risk of malnutrition defined according to the modified Global Leadership Initiative on Malnutrition (GLIM) criteria in men (OR 0.59, 95% CI 0.41 to 0.86). However, the same study did not find an association between the MedDiet and malnutrition [89]. For cohort study, the Health, Aging, and Body Composition (Health ABC) Study found that a poor diet quality as assessed using the HEI was not associated with 4-year incidence of protein-energy malnutrition (PEM) and 3-year incidence of persistent PEM among 2234 US community-dwelling older adults [90]. 

Adiposity can be considered as a proxy for nutritional status. Using the data of 508 US older adults in the National Health and Nutrition Examination Surveys (NHANES), it was shown that adherence to the DASH diet was inversely associated with visceral adiposity index (a reliable estimate of visceral adiposity which incorporates waist circumference, body mass index, circulating triglycerides, and high-density lipoprotein cholesterol data) [91]. In the Multi-Ethnic Study of Atherosclerosis (MESA) cohort, a cross-sectional analysis of 5079 older adults showed that a higher diet quality score (composed of features consistent with the American Heart Association goals and a Mediterranean-style diet) was associated with lower visceral fat obtained by computed tomography [92]. A cross-sectional study of 829 middle-aged and older Japanese adults from the Waseda Alumni’s Sports, Exercise, Daily Activity, Sedentariness, and Health Study (WASEDA’s Health Study) showed that “healthy Japanese” dietary pattern (high intake of vegetables and fruits, soy products, mushrooms, seaweeds and fish) was inversely associated with visceral fat in men only, whereas the “seafood and alcohol” dietary pattern was not associated with visceral fat measured using magnetic resonance imaging [93]. 

To the best of our knowledge, only one study examined the association between dietary patterns and cardiorespiratory fitness in older adults. Data from 1235 community-dwelling Chinese older adults showed that a higher Okinawan diet score was associated with a higher 7-year cardiorespiratory fitness in men only, whereas none of the dietary pattern scores including DQI-I, Okinawan, DASH, MedDiet, or MIND diets were associated with peak oxygen uptake in women [94]. 

Vitality has also been operationalized as the absence of deficits in the components of frailty assessment [95]. Components of frailty including fatigue, decline in strength and aerobic function, weight loss, and multi-morbidity are highly related to nutrition [96]. Systematic review and meta-analysis have shown the inverse association between adherence to the MedDiet and frailty risk [97,98,99]. However, the Mediterranean-style dietary pattern was not associated with frailty in Japanese and Chinese older adults [100,101]. The different findings may indicate the differences between the “western” and “eastern” MedDiet. Several healthy dietary patterns have also been found to be associated with frailty. For example, a better diet quality as assessed by the DQI-I was associated with lower odds of developing frailty during 4-year follow-up among 2724 Chinese community-dwelling older adults [100]. Similarly, the Rotterdam Study found that adherence to the Dutch Healthy Diet (high intakes of vegetables, fruits, fiber, fish, and fish oil; low intakes of saturated fatty acid, trans-fatty acids, sodium, and alcohol) was associated with lower frailty scores over 4 years among 2253 older adults [102]. In the Health ABC Study of 2154 older adults, it was found that consumption of poor- (hazard ratio (HR) 1.92, 95% CI 1.17 to 3.17) and medium-quality diets (HR 1.40, 95% CI 0.99 to 1.98) as assessed with the HEI was associated with higher 4-year incidence of frailty compared with consumption of good-quality diets [103]. The association between the inflammatory potential of diet on frailty has also been demonstrated. In the Seniors-ENRICA study of 1915 older adults, those in the highest tertile of DII showed a higher risk of frailty at 3-years follow-up (OR 2.48, 95% CI 1.42 to 1.44) compared with those in the lowest tertile of DII [104]. Regarding the association between posteriori dietary patterns and frailty, a prospective study of 945 older men from the British Regional Heart Study found that those who followed a prudent diet (high in poultry, fish, vegetables, legumes, pasta and rice, and eggs) were less likely to become frail (OR 0.47, 95% 0.26 to 0.85) and those who had a high-fat/low-fiber diet pattern were more likely to become frail (OR 2.05, 95% CI 1.15 to 3.62) at 3-year of follow up [105]. The Rotterdam Study found that adherence to the Dutch “traditional” dietary pattern (high intake of savory snacks, legumes, eggs, fried potatoes, alcohol, processed meat, and soup) was associated with a lower risk of frailty over 4 years. In contrast, “carnivore” (high intakes of red meat and poultry; low intake of meat replacements) and “health-conscious” (high intakes of whole grains, vegetables, fruit, and nuts) dietary patterns were not associated with risk of frailty [102]. For a longer follow-up period, the Three-City Study Bordeaux cohort of 972 older adults showed that a “healthy” dietary pattern (high intake of fish in men and fruits and vegetables in women) was associated with a lower risk of developing frailty during a follow-up of 12 years. Conversely, greater adherence to the “pasta” pattern in men and the “biscuits and snacking” pattern in women was associated with a higher risk of frailty compared with the “healthy” pattern. However, the “meat and alcohol”, “charcuterie and starchy foods”, and “pizza and sandwiches” patterns were not associated with frailty [106]. Among Asian countries, the Nagoya Longitudinal Study for Healthy Elderly study (NLS-HE) found that adherence to “salt and pickles” and “sugar and fat” dietary patterns were positively associated with frailty over 3 years, whereas adherence to “protein-rich” dietary pattern plays a beneficial role in frailty prevention among community-dwelling Japanese older adults [101]. Among Chinese community-dwelling older adults, posteriori dietary patterns, namely “vegetables-fruits”, “snacks-drinks-milk products”, and “meat-fish” were not associated with 4-year incident frailty [100]. 

### 2.5. Locomotion

Mobility is an essential determinant for healthy aging. An individual’s bodily capacity to move from one place to another is termed locomotor capacity [87]. Mobility limitation is common among older adults and is associated with loss of strength and/or function that characterizes sarcopenia [107]. A systematic review included five studies that investigated the prospective association between the MedDiet and physical performance in 6040 community-dwelling older adults [23]. The pooled analysis indicated that high adherence to the MedDiet was not associated with a reduced decline in physical performance as assessed using walking speed, handgrip strength, Short Physical Performance Battery (SPPB), or self-reported agility, mobility, and overall physical performance. Similarly, the Nagoya Longitudinal Study for Healthy Elderly cohort found that adherence to the MedDiet was not associated with changes in walking speed, handgrip strength, and skeletal muscle mass during 3-year of follow-up in 666 older adults [108]. Furthermore, a prospective analysis among 2948 Chinese community-dwelling older adults did not find an association between the MedDiet and incident sarcopenia at 4-years [109]. 

Other dietary patterns have been shown to be protective for locomotor capacity. Among 282 older women from the control group of the Osteoporosis Risk Factor and Prevention-Fracture Prevention Study (OSTPRE-FPS), those in the highest Baltic Sea Diet (BSD) score quartile showed the highest SPPB improvement, having a 55% higher squat test completion and 67% lower risk of sarcopenia as compared to the lowest quartile over 3-years of follow-up [110]. Similarly, in the Helsinki Birth Cohort Study among 1072 older adults, women with a higher BSD score had on average five points higher Senior Fitness Test score, 17% better result in the 6-min walking test, and 20% better chair stand results compared with those with a lower BSD score at 10-year follow-up. Such associations were not observed in men [111]. In the same cohort, it was found that handgrip strength was on average 5% and leg strength 7% greater at 10-year follow-up among women who had higher BSD score compared to those with lower BSD score, whereas no association between healthy Nordic diet and muscle strength was observed among men [112]. Data from 1234 men from the British Regional Heart Study showed that men with greater adherence to the Elderly Dietary Index (EDI) or Healthy Diet Indicator (HDI) were less likely to have self-reported mobility limitation 15 years later [113]. Although the Seniors-ENRICA study did not find any association between DII and low physical performance as assessed with SPPB at 3-year follow-up, it was found that a higher DII score (higher inflammatory potential of diet) was associated with slow gait speed, one of the components of the SPPB and Fried’s frailty criterion [104]. A similar result was found in the cross-sectional analysis of the Kashiwa-based study, men with the higher energy-adjusted DII score (a diet with pro-inflammatory potential) showed a 2.89-times higher risk of sarcopenia than those with a lower score [114]. 

Association between posteriori dietary patterns and locomotor capacity has also been explored. In the Three-City Study Bordeaux cohort of 583 French older community-dwelling older adults, men in the “biscuits and snacking” pattern were at higher risk of self-reported mobility restriction compared with those belonging to the “healthy” pattern. Among women, none of the posteriori dietary patterns (“healthy”, “small eaters”, “biscuits, and snacking”, “charcuterie, starchy foods”, and “pizza, sandwich”) were associated with the risk of self-reported mobility restriction during a mean of 9 years of follow-up [115]. Similarly, the British Regional Heart study showed that men with a higher score for the “high-fat/low-fiber” pattern (high consumption of red meat, meat products, fried potato, white bread, eggs, and beer; and low intake of wholemeal bread) at baseline were more likely to have self-reported mobility limitation at 15-year follow-up [113]. Among the very old (aged 85+), the Newcastle 85+ Study showed that older adults in the “high red meat” pattern (high in red meats/meat dishes, gravy, and potato) or “high butter” pattern (high in butter and low in unsaturated fats) were associated with worse overall handgrip strength and Timed Up-and Go performance over 5 years [116]. In the same cohort, data of 757 older adults found that those in the “traditional British” pattern (high in butter, red meat, gravy, and potatoes) was associated with an increased risk of sarcopenia at 3-year follow-up regardless of protein status compared with “low butter” pattern [117]. Various cross-sectional studies have demonstrated consistent results regarding the associations between (un)healthy posteriori dietary patterns and locomotor capacity: adherence to Western dietary pattern and slower gait speed and worse 30 s chair stand test performance in Spanish [118]; “coarse cereals and vegetables” pattern and “animal food” pattern and lower prevalence of sarcopenia in Chinese [119,120]; “traditional Japanese” dietary pattern (high intake of fish, soybean products, vegetables, and fruits) [121] and dietary pattern characterized by high intakes of fish, soybean, potatoes, most vegetables, mushrooms, seaweeds, and fruit and low intake of rice, and lower prevalence of sarcopenia in Japanese [122]. 

### 2.6. Multidimensional Concepts of Healthy Aging

It should be acknowledged that the five domains of intrinsic capacity closely interact with the others. Instead of focusing on specific diseases or functions of the body, a multidimensional concept of healthy aging has been developed to consider the overall health of individuals during aging [4]. To the best of our knowledge, only one nutritional epidemiological study used the concept of intrinsic capacity as an outcome measure. The NLS-HE study examined the association between dietary patterns and changes in intrinsic capacity over 3 years among 666 community-dwelling Japanese older adults. The findings showed that adherence to the “fruits and vegetables” and “protein-rich” (animal-based protein in particular) dietary patterns were associated with greater intrinsic capacity, whereas adherence to the “sugar and fat” dietary pattern was associated with decreased intrinsic capacity [123]. 

Most available studies used their own definitions to define healthy aging. In the Blue Mountains Eye Study (BMES), successful aging was defined as the absence of disability, depressive symptoms, cognitive impairment, respiratory symptoms, and three chronic diseases. Among 1609 middle-aged adults who completed the 10-year follow-up, those with a higher adherence to the Australian dietary guidelines at baseline had 58% higher odds of successful aging after 10 years compared with those with a lower adherence [124]. The SU.VI.MAX study defined healthy aging as having good physical, cognitive and social functioning, good overall self-perceived health, independence in instrumental activities of daily living, combined with the absence of incident major chronic disease, depressive symptoms and function-limiting pain. The study showed that higher adherence to the MedDiet at midlife was associated with a nearly 40% higher odds of healthy aging during a mean follow-up of 13.4 years in French adults [125]. Furthermore, data of 10,670 women from the Nurses’ Health Study showed that greater adherence to the alternate MedDiet and alternative HEI-2020 at midlife was associated with approximately 40% greater odds of healthy aging (defined as the absence of 11 chronic diseases and impairments in cognitive, physical function or mental health) after an average of 15 years [126]. In the Singapore Chinese Health Study, healthy aging was similarly defined as the absence of 10 chronic diseases, absence of cognitive impairment and limitations in instrumental activities of daily living, good mental and overall self-perceived health, good physical functioning, and no function-limiting pain. It was found that higher scores of healthy dietary patterns at midlife, including the alternate MedDiet, the DASH diet, the alternative HEI-2010, overall plant-based diet index (PDI), and healthful plant-based diet index (hPDI), were associated with a higher likelihood of healthy aging at about 20 years after the baseline visit. Each standard deviation increment in different dietary pattern scores was associated with 12% to 18% higher likelihood of healthy aging [127]. 

Several posteriori dietary patterns have also been found to be associated with multidimensional concepts of healthy aging. Among 6308 older adults from the Melbourne Collaborative Cohort Study, adherence to the “fruit” pattern was positively associated with successful aging and adherence to the “meat/fatty” pattern was inversely associated with successful aging (defined as the absence of major chronic diseases, and major limitations of physical function while maintaining good mental health) at a mean of 11.7-year of follow up [128]. Data from the Whitehall II study of 5350 middle-aged adults showed that participants with a “Western-type” dietary pattern (characterized by high intakes of fried and sweet food, processed food and red meat, refined grains, and high-fat dairy products) had lower odds of ideal aging (free of chronic conditions and high performance in physical, mental, and cognitive functioning test) at a mean of 16-year of follow-up. However, there was no significant association between “healthy-foods” dietary pattern (characterized by high intake of vegetables, fruits, and fish) and ideal aging [129]. 

## 3. Concluding Remarks and Future Directions

Is there a consensus on the definition of a healthy diet for healthy aging? Although the evidence on healthy aging differs by different dietary patterns, it appears that dietary patterns centered on plant-based foods have a beneficial role in (domains of) healthy aging. These healthy dietary patterns, either priori or posteriori, are composed of common dietary components, including high consumption of fruits, vegetables, and whole grains; moderate consumption of dairy products, fish, and poultry; and low consumption of sugars, saturated fat, and processed foods. Although it is outside the scope of this review to address the methodological issues of the included studies in detail, it should be noted that the region in which the study was conducted and the method used to define dietary patterns may give more insights to define a healthy diet for healthy aging. After all, factors such as culture, ethnicity, geographical locations, and cooking methods may affect the associations. In this context, dietary guidelines should be culturally adapted to achieve higher adherence and better outcomes. While the underlying mechanisms for the role of diet on healthy aging differ by the dietary patterns, these mainly include anti-inflammatory and anti-oxidant properties to combat oxidative stress, which has been considered an important factor in the physiopathology of aging [130,131]. 

Despite the ample studies supporting the association between healthy dietary patterns and (domains of) healthy aging, one should keep in mind that diet is one of the components of a healthy lifestyle. Other components such as regular physical activity should be recommended together with dietary advice. It should be noted that most studies were conducted in middle-aged and older adults with normal (physical/cognitive) functions. Whether adherence to these dietary patterns might be beneficial for healthy aging in a population with suboptimal functions remains to be established. These also emphasize the importance of a life-course approach, i.e., promote a healthy diet as early as possible for healthy aging, as evidence has shown that healthy aging is fostered by the cumulative effects of healthy nutrition early in life [132]. 

Our review identified a knowledge gap in the area of dietary patterns and multidimensional concepts of healthy aging. Most of the current evidence is based on a specific domain of healthy aging. To the best of our knowledge, only one study examined dietary patterns and healthy aging using the concept of intrinsic capacity [123]. Other relevant studies considered the absence of major chronic diseases as one of the components to define healthy aging [124,125,126,127,128,129]. This concept is different from the definition of healthy aging proposed by the WHO, which considers health from a functional rather than disease-based perspective [5]. More epidemiological studies regarding dietary patterns and multidimensional concepts of healthy aging are warranted and should consider intrinsic capacity as an outcome measure to further our understanding of a healthy diet for healthy aging. For now, the causal effects of healthy dietary patterns on healthy aging are not well-studied, as the majority of the findings come from epidemiological studies and trials of dietary interventions are limited and merely focused on one specific domain of healthy aging. When a sufficient number of epidemiological studies is available, evidence of dietary patterns and multidimensional concepts of healthy aging can be synthesized. This will help to inform the design of clinical trials evaluating a healthy diet as a strategy for healthy aging to further our progress in translating evidence to practice and promoting healthy aging. When promising results from clinical trials become available, the next essential step would be to explore strategies to achieve a sustained change in dietary behaviors in the real world and to create an environment to make healthy eating affordable and accessible. 

## Figures and Tables

**Table 1:** Characteristics of common priori dietary patterns examined in the literature.

Dietary Patterns	Characteristics
Mediterranean diet (MedDiet) [14]	Rich in fruits, vegetables, whole grains, olive oil, nuts, legumes; moderate intake of fish and other meat, dairy products and red wine; and low intakes of eggs and sweets
Dietary Approach to Stop Hypertension (DASH) [17]	Rich in fruits, vegetables, fish, whole grains, nuts and legumes, low-fat dairy products; limited in fats, saturated fat, cholesterol, red and processed meats, sweets and sugar-containing beverages
Mediterranean-DASH diet Intervention for Neurodegenerative Delay (MIND) [18]	Consists of 10 brain-healthy food groups (green leafy vegetables, other vegetables, nuts, berries, beans, whole grains, seafood, poultry, olive oil, and wine) and 5 unhealthy food groups (red meats, butter and stick margarine, cheese, pastries and sweets, and fried/fast foods)
Baltic Sea diet [19]	High intakes of total fruits (mostly apples and pears) and berries, vegetables, cereal (rye, oats and barley), fish, low-fat milk, and a high polyunsaturated fatty acid: saturated fatty acid ratio; limit processed meat products, percent energy from fat, and alcohol intake
Healthy Eating Index (HEI) [12]	Conforms to the serving recommendations of the USDA Food Guide Pyramid for five major food groups: grains, vegetables, fruits, milk, and meat; overall intake of fat, saturated fat, cholesterol, and sodium; and amount of variety in an individual’s diet.
Okinawan diet [15]	Moderate caloric restriction; high consumption of vegetables (particularly root and green-yellow vegetables) and legumes (mostly soybean); regular consumption of fish and seafood; low consumption of meat products (mostly lean pork), dairy products and fat; emphasis on low glycemic index carbohydrates, and moderate alcohol consumption

## Data Availability

Data sharing not applicable.

## References

[1] Jivraj S., Goodman A., Pongiglione B., Ploubidis G.B. (2020). Living longer but not necessarily healthier: The joint progress of health and mortality in the working-age population of england. Popul. Stud..

[2] World Health Assembly (2016). The Global Strategy and Action Plan on Ageing and Health 2016–2020: Towards a World in Which Everyone Can Live a Long and Healthy Life.

[3] Olshansky S.J. (2018). From lifespan to healthspan. JAMA.

[4] World Health Organization (2017). Integrated Care for Older People: Guidelines on Community-Level Interventions to Manage Declines in Intrinsic Capacity.

[5] Beard J.R., Officer A., de Carvalho I.A., Sadana R., Pot A.M., Michel J.P., Lloyd-Sherlock P., Epping-Jordan J.E., Peeters G., Mahanani W.R. (2016). The world report on ageing and health: A policy framework for healthy ageing. Lancet.

[6] Cesari M., Araujo de Carvalho I., Amuthavalli Thiyagarajan J., Cooper C., Martin F.C., Reginster J.Y., Vellas B., Beard J.R. (2018). Evidence for the domains supporting the construct of intrinsic capacity. J. Gerontol. A Biol. Sci. Med. Sci..

[7] Stuck A.E., Siu A.L., Wieland G.D., Adams J., Rubenstein L.Z. (1993). Comprehensive geriatric assessment: A meta-analysis of controlled trials. Lancet.

[8] Kiefte-de Jong J.C., Mathers J.C., Franco O.H. (2014). Nutrition and healthy ageing: The key ingredients. Proc. Nutr. Soc..

[9] Jacques P.F., Tucker K.L. (2001). Are dietary patterns useful for understanding the role of diet in chronic disease?. Am. J. Clin. Nutr..

[10] Schulze M.B., Martinez-Gonzalez M.A., Fung T.T., Lichtenstein A.H., Forouhi N.G. (2018). Food based dietary patterns and chronic disease prevention. BMJ.

[11] Zhao J., Li Z., Gao Q., Zhao H., Chen S., Huang L., Wang W., Wang T. (2021). A review of statistical methods for dietary pattern analysis. Nutr. J..

[12] Kennedy E.T., Ohls J., Carlson S., Fleming K. (1995). The healthy eating index—Design and applications. J. Am. Diet. Assoc..

[13] Harsha D.W., Lin P.H., Obarzanek E., Karanja N.M., Moore T.J., Caballero B. (1999). Dietary approaches to stop hypertension: A summary of study results. Dash collaborative research group. J. Am. Diet. Assoc..

[14] Davis C., Bryan J., Hodgson J., Murphy K. (2015). Definition of the mediterranean diet; a literature review. Nutrients.

[15] Willcox B.J., Willcox D.C., Todoriki H., Fujiyoshi A., Yano K., He Q., Curb J.D., Suzuki M. (2007). Caloric restriction, the traditional okinawan diet, and healthy aging: The diet of the world’s longest-lived people and its potential impact on morbidity and life span. Ann. N. Y. Acad. Sci..

[16] Panagiotakos D. (2008). A-priori versus α-posterior methods in dietary pattern analysis: A review in nutrition epidemiology. Nutr. Bull..

[17] Sacks F.M., Svetkey L.P., Vollmer W.M., Appel L.J., Bray G.A., Harsha D., Obarzanek E., Conlin P.R., Miller E.R., Simons-Morton D.G. (2001). Effects on blood pressure of reduced dietary sodium and the dietary approaches to stop hypertension (dash) diet. N. Engl. J. Med..

[18] Marcason W. (2015). What are the components to the mind diet?. J. Acad. Nutr. Diet..

[19] Kanerva N., Kaartinen N.E., Schwab U., Lahti-Koski M., Mannisto S. (2014). The baltic sea diet score: A tool for assessing healthy eating in nordic countries. Public Health Nutr..

[20] Harada C.N., Natelson Love M.C., Triebel K.L. (2013). Normal cognitive aging. Clin. Geriatr. Med..

[21] Rajaram S., Jones J., Lee G.J. (2019). Plant-based dietary patterns, plant foods, and age-related cognitive decline. Adv. Nutr..

[22] van den Brink A.C., Brouwer-Brolsma E.M., Berendsen A.A.M., van de Rest O. (2019). The mediterranean, dietary approaches to stop hypertension (dash), and mediterranean-dash intervention for neurodegenerative delay (mind) diets are associated with less cognitive decline and a lower risk of alzheimer’s disease-a review. Adv. Nutr..

[23] Coelho-Júnior H.J., Trichopoulou A., Panza F. (2021). Cross-sectional and longitudinal associations between adherence to mediterranean diet with physical performance and cognitive function in older adults: A systematic review and meta-analysis. Ageing Res. Rev..

[24] McEvoy C.T., Hoang T., Sidney S., Steffen L.M., Jacobs D.R., Shikany J.M., Wilkins J.T., Yaffe K. (2019). Dietary patterns during adulthood and cognitive performance in midlife: The cardia study. Neurology.

[25] Radd-Vagenas S., Duffy S.L., Naismith S.L., Brew B.J., Flood V.M., Fiatarone Singh M.A. (2018). Effect of the mediterranean diet on cognition and brain morphology and function: A systematic review of randomized controlled trials. Am. J. Clin. Nutr..

[26] Valls-Pedret C., Sala-Vila A., Serra-Mir M., Corella D., de la Torre R., Martinez-Gonzalez M.A., Martinez-Lapiscina E.H., Fito M., Perez-Heras A., Salas-Salvado J. (2015). Mediterranean diet and age-related cognitive decline: A randomized clinical trial. JAMA Intern. Med..

[27] Lee J., Pase M., Pipingas A., Raubenheimer J., Thurgood M., Villalon L., Macpherson H., Gibbs A., Scholey A. (2015). Switching to a 10-day mediterranean-style diet improves mood and cardiovascular function in a controlled crossover study. Nutrition.

[28] Martinez-Lapiscina E.H., Clavero P., Toledo E., Estruch R., Salas-Salvado J., San Julian B., Sanchez-Tainta A., Ros E., Valls-Pedret C., Martinez-Gonzalez M.A. (2013). Mediterranean diet improves cognition: The predimed-navarra randomised trial. J. Neurol. Neurosurg. Psychiatry.

[29] Marseglia A., Xu W., Fratiglioni L., Fabbri C., Berendsen A.A.M., Bialecka-Debek A., Jennings A., Gillings R., Meunier N., Caumon E. (2018). Effect of the nu-age diet on cognitive functioning in older adults: A randomized controlled trial. Front. Physiol..

[30] Knight A., Bryan J., Wilson C., Hodgson J.M., Davis C.R., Murphy K.J. (2016). The mediterranean diet and cognitive function among healthy older adults in a 6-month randomised controlled trial: The medley study. Nutrients.

[31] Davis C.R., Bryan J., Hodgson J.M., Wilson C., Murphy K.J. (2015). Older australians can adhere to a traditional mediterranean style diet over two weeks: A pilot dietary intervention study. BMC Nutr..

[32] Wade A.T., Davis C.R., Dyer K.A., Hodgson J.M., Woodman R.J., Keage H.A.D., Murphy K.J. (2019). A mediterranean diet with fresh, lean pork improves processing speed and mood: Cognitive findings from the medpork randomised controlled trial. Nutrients.

[33] Haring B., Wu C., Mossavar-Rahmani Y., Snetselaar L., Brunner R., Wallace R.B., Neuhouser M.L., Wassertheil-Smoller S. (2016). No association between dietary patterns and risk for cognitive decline in older women with 9-year follow-up: Data from the women’s health initiative memory study. J. Acad. Nutr. Diet..

[34] Berendsen A.A.M., Kang J.H., van de Rest O., Feskens E.J.M., de Groot L., Grodstein F. (2017). The dietary approaches to stop hypertension diet, cognitive function, and cognitive decline in american older women. J. Am. Med. Dir. Assoc..

[35] Morris M.C., Tangney C.C., Wang Y., Sacks F.M., Barnes L.L., Bennett D.A., Aggarwal N.T. (2015). Mind diet slows cognitive decline with aging. Alzheimers Dement..

[36] Berendsen A.M., Kang J.H., Feskens E.J.M., de Groot C., Grodstein F., van de Rest O. (2018). Association of long-term adherence to the mind diet with cognitive function and cognitive decline in american women. J. Nutr. Health Aging.

[37] Hosking D.E., Eramudugolla R., Cherbuin N., Anstey K.J. (2019). Mind not mediterranean diet related to 12-year incidence of cognitive impairment in an australian longitudinal cohort study. Alzheimers Dement..

[38] Shakersain B., Rizzuto D., Larsson S.C., Faxen-Irving G., Fratiglioni L., Xu W.L. (2018). The nordic prudent diet reduces risk of cognitive decline in the swedish older adults: A population-based cohort study. Nutrients.

[39] Shivappa N., Steck S.E., Hurley T.G., Hussey J.R., Hebert J.R. (2014). Designing and developing a literature-derived, population-based dietary inflammatory index. Public Health Nutr..

[40] Hayden K.M., Beavers D.P., Steck S.E., Hebert J.R., Tabung F.K., Shivappa N., Casanova R., Manson J.E., Padula C.B., Salmoirago-Blotcher E. (2017). The association between an inflammatory diet and global cognitive function and incident dementia in older women: The women’s health initiative memory study. Alzheimers Dement..

[41] Kesse-Guyot E., Assmann K.E., Andreeva V.A., Touvier M., Neufcourt L., Shivappa N., Hebert J.R., Wirth M.D., Hercberg S., Galan P. (2017). Long-term association between the dietary inflammatory index and cognitive functioning: Findings from the su.Vi.Max study. Eur. J. Nutr..

[42] Abbatecola A.M., Russo M., Barbieri M. (2018). Dietary patterns and cognition in older persons. Curr. Opin. Clin. Nutr. Metab. Care.

[43] Ozawa M., Shipley M., Kivimaki M., Singh-Manoux A., Brunner E.J. (2017). Dietary pattern, inflammation and cognitive decline: The whitehall ii prospective cohort study. Clin. Nutr..

[44] Chen Y.C., Jung C.C., Chen J.H., Chiou J.M., Chen T.F., Chen Y.F., Tang S.C., Yeh S.J., Lee M.S. (2017). Association of dietary patterns with global and domain-specific cognitive decline in chinese elderly. J. Am. Geriatr. Soc..

[45] World Health Organization (2017). Depression and Other Common Mental Disorders: Global Health Estimates.

[46] Chu W., Chang S.F., Ho H.Y., Lin H.C. (2019). The relationship between depression and frailty in community-dwelling older people: A systematic review and meta-analysis of 84,351 older adults. J. Nurs. Sch..

[47] Sivertsen H., Bjorklof G.H., Engedal K., Selbaek G., Helvik A.S. (2015). Depression and quality of life in older persons: A review. Dement. Geriatr. Cogn. Disord..

[48] Valiengo Lda C., Stella F., Forlenza O.V. (2016). Mood disorders in the elderly: Prevalence, functional impact, and management challenges. Neuropsychiatr. Dis. Treat..

[49] Huang Q., Liu H., Suzuki K., Ma S., Liu C. (2019). Linking what we eat to our mood: A review of diet, dietary antioxidants, and depression. Antioxidants.

[50] Marx W., Lane M., Hockey M., Aslam H., Berk M., Walder K., Borsini A., Firth J., Pariante C.M., Berding K. (2021). Diet and depression: Exploring the biological mechanisms of action. Mol. Psychiatry.

[51] Lassale C., Batty G.D., Baghdadli A., Jacka F., Sanchez-Villegas A., Kivimaki M., Akbaraly T. (2019). Healthy dietary indices and risk of depressive outcomes: A systematic review and meta-analysis of observational studies. Mol. Psychiatry.

[52] Tolkien K., Bradburn S., Murgatroyd C. (2019). An anti-inflammatory diet as a potential intervention for depressive disorders: A systematic review and meta-analysis. Clin. Nutr..

[53] Wang J., Zhou Y., Chen K., Jing Y., He J., Sun H., Hu X. (2018). Dietary inflammatory index and depression: A meta-analysis. Public Health Nutr.

[54] Ljungberg T., Bondza E., Lethin C. (2020). Evidence of the importance of dietary habits regarding depressive symptoms and depression. Int. J. Environ. Res. Public Health.

[55] Wu P.Y., Chen K.M., Belcastro F. (2021). Dietary patterns and depression risk in older adults: Systematic review and meta-analysis. Nutr. Rev..

[56] Cherian L., Wang Y., Holland T., Agarwal P., Aggarwal N., Morris M.C. (2021). Dash and mediterranean-dash intervention for neurodegenerative delay (mind) diets are associated with fewer depressive symptoms over time. J. Gerontol. A Biol. Sci. Med. Sci..

[57] Das A., Cumming R.G., Naganathan V., Ribeiro R.V., Le Couteur D.G., Handelsman D.J., Waite L.M., Hirani V. (2021). The association between antioxidant intake, dietary pattern and depressive symptoms in older australian men: The concord health and ageing in men project. Eur. J. Nutr..

[58] Gianfredi V., Koster A., Odone A., Amerio A., Signorelli C., Schaper N.C., Bosma H., Kohler S., Dagnelie P.C., Stehouwer C.D.A. (2021). Associations of dietary patterns with incident depression: The maastricht study. Nutrients.

[59] Tsai H.J. (2016). Dietary patterns and depressive symptoms in a taiwanese population aged 53 years and over: Results from the taiwan longitudinal study of aging. Geriatr. Gerontol. Int..

[60] Okubo R., Matsuoka Y.J., Sawada N., Mimura M., Kurotani K., Nozaki S., Shikimoto R., Tsugane S. (2019). Diet quality and depression risk in a japanese population: The japan public health center (jphc)-based prospective study. Sci. Rep..

[61] Hart M.J., Milte C.M., Torres S.J., Thorpe M.G., McNaughton S.A. (2019). Dietary patterns are associated with depressive symptoms in older australian women but not men. Br. J. Nutr..

[62] Liao K., Gu Y., Liu M., Fu J., Wang X., Yang G., Zhang Q., Liu L., Meng G., Yao Z. (2019). Association of dietary patterns with depressive symptoms in chinese postmenopausal women. Br. J. Nutr..

[63] Vermeulen E., Stronks K., Visser M., Brouwer I.A., Schene A.H., Mocking R.J., Colpo M., Bandinelli S., Ferrucci L., Nicolaou M. (2016). The association between dietary patterns derived by reduced rank regression and depressive symptoms over time: The invecchiare in chianti (inchianti) study. Br. J. Nutr..

[64] Yokoyama Y., Kitamura A., Yoshizaki T., Nishi M., Seino S., Taniguchi Y., Amano H., Narita M., Shinkai S. (2019). Score-based and nutrient-derived dietary patterns are associated with depressive symptoms in community-dwelling older japanese: A cross-sectional study. J. Nutr. Health Aging.

[65] Wade A.T., Davis C.R., Dyer K.A., Hodgson J.M., Woodman R.J., Keage H.A.D., Murphy K.J. (2020). A mediterranean diet supplemented with dairy foods improves mood and processing speed in an australian sample: Results from the meddairy randomized controlled trial. Nutr. Neurosci..

[66] Sanchez-Villegas A., Martinez-Gonzalez M.A., Estruch R., Salas-Salvado J., Corella D., Covas M.I., Aros F., Romaguera D., Gomez-Gracia E., Lapetra J. (2013). Mediterranean dietary pattern and depression: The predimed randomized trial. BMC Med..

[67] Burton M.J., Ramke J., Marques A.P., Bourne R.R.A., Congdon N., Jones I., Ah Tong B.A.M., Arunga S., Bachani D., Bascaran C. (2021). The lancet global health commission on global eye health: Vision beyond 2020. Lancet Glob. Health.

[68] Hong T., Mitchell P., Burlutsky G., Samarawickrama C., Wang J.J. (2014). Visual impairment and the incidence of falls and fractures among older people: Longitudinal findings from the blue mountains eye study. Invest. Ophthalmol. Vis. Sci..

[69] Zetterlund C., Lundqvist L.O., Richter H.O. (2019). Visual, musculoskeletal and balance symptoms in individuals with visual impairment. Clin. Exp. Optom..

[70] Francisco S.G., Smith K.M., Aragones G., Whitcomb E.A., Weinberg J., Wang X., Bejarano E., Taylor A., Rowan S. (2020). Dietary patterns, carbohydrates, and age-related eye diseases. Nutrients.

[71] Chiu C.J., Chang M.L., Li T., Gensler G., Taylor A. (2017). Visualization of dietary patterns and their associations with age-related macular degeneration. Invest. Ophthalmol. Vis. Sci..

[72] de Koning-Backus A.P.M., Buitendijk G.H.S., Kiefte-de Jong J.C., Colijn J.M., Hofman A., Vingerling J.R., Haverkort E.B., Franco O.H., Klaver C.C.W. (2019). Intake of vegetables, fruit, and fish is beneficial for age-related macular degeneration. Am. J. Ophthalmol..

[73] Merle B.M.J., Colijn J.M., Cougnard-Gregoire A., de Koning-Backus A.P.M., Delyfer M.N., Kiefte-de Jong J.C., Meester-Smoor M., Feart C., Verzijden T., Samieri C. (2019). Mediterranean diet and incidence of advanced age-related macular degeneration: The eye-risk consortium. Ophthalmology.

[74] Raimundo M., Mira F., Cachulo M.D.L., Barreto P., Ribeiro L., Farinha C., Lains I., Nunes S., Alves D., Figueira J. (2018). Adherence to a mediterranean diet, lifestyle and age-related macular degeneration: The coimbra eye study—Report 3. Acta. Ophthalmol..

[75] Nunes S., Alves D., Barreto P., Raimundo M., da Luz Cachulo M., Farinha C., Lains I., Rodrigues J., Almeida C., Ribeiro L. (2018). Adherence to a mediterranean diet and its association with age-related macular degeneration. The coimbra eye study-report 4. Nutrition.

[76] Keenan T.D., Agron E., Mares J., Clemons T.E., van Asten F., Swaroop A., Chew E.Y., Age-Related Eye Disease S., Research G. (2020). Adherence to the mediterranean diet and progression to late age-related macular degeneration in the age-related eye disease studies 1 and 2. Ophthalmology.

[77] Dighe S., Zhao J., Steffen L., Mares J.A., Meuer S.M., Klein B.E.K., Klein R., Millen A.E. (2020). Diet patterns and the incidence of age-related macular degeneration in the atherosclerosis risk in communities (aric) study. Br. J. Ophthalmol..

[78] Garcia-Layana A., Ciufo G., Toledo E., Martinez-Gonzalez M.A., Corella D., Fito M., Estruch R., Gomez-Gracia E., Fiol M., Lapetra J. (2017). The effect of a mediterranean diet on the incidence of cataract surgery. Nutrients.

[79] Diaz-Lopez A., Babio N., Martinez-Gonzalez M.A., Corella D., Amor A.J., Fito M., Estruch R., Aros F., Gomez-Gracia E., Fiol M. (2015). Mediterranean diet, retinopathy, nephropathy, and microvascular diabetes complications: A post hoc analysis of a randomized trial. Diabetes Care.

[80] Deal J.A., Betz J., Yaffe K., Harris T., Purchase-Helzner E., Satterfield S., Pratt S., Govil N., Simonsick E.M., Lin F.R. (2017). Hearing impairment and incident dementia and cognitive decline in older adults: The health abc study. J. Gerontol. A Biol. Sci. Med. Sci..

[81] Cosh S., Helmer C., Delcourt C., Robins T.G., Tully P.J. (2019). Depression in elderly patients with hearing loss: Current perspectives. Clin. Interv Aging.

[82] Martinez-Amezcua P., Powell D., Kuo P.-L., Reed N.S., Sullivan K.J., Palta P., Szklo M., Sharrett R., Schrack J.A., Lin F.R. (2021). Association of age-related hearing impairment with physical functioning among community-dwelling older adults in the us. JAMA Netw. Open.

[83] Curhan S.G., Halpin C., Wang M., Eavey R.D., Curhan G.C. (2020). Prospective study of dietary patterns and hearing threshold elevation. Am. J. Epidemiol..

[84] Croll P.H., Voortman T., Vernooij M.W., Baatenburg de Jong R.J., Lin F.R., Rivadeneira F., Ikram M.A., Goedegebure A. (2019). The association between obesity, diet quality and hearing loss in older adults. Aging.

[85] Gallagher N.E., Patterson C.C., Neville C.E., Yarnell J., Ben-Shlomo Y., Fehily A., Gallacher J.E., Lyner N., Woodside J.V. (2019). Dietary patterns and hearing loss in older men enrolled in the caerphilly study. Br. J. Nutr..

[86] Dawes P., Cruickshanks K.J., Marsden A., Moore D.R., Munro K.J. (2020). Relationship between diet, tinnitus, and hearing difficulties. Ear Hear..

[87] World Health Organization (2019). Integrated Care for Older People (Icope): Guidance for Person-Centred Assessment and Pathways in Primary Care.

[88] Katsas K., Mamalaki E., Kontogianni M.D., Anastasiou C.A., Kosmidis M.H., Varlamis I., Hadjigeorgiou G.M., Dardiotis E., Sakka P., Scarmeas N. (2020). Malnutrition in older adults: Correlations with social, diet-related, and neuropsychological factors. Nutrition.

[89] Yeung S.S.Y., Chan R.S.M., Lee J.S.W., Woo J. (2021). Certain dietary patterns are associated with glim criteria among chinese community-dwelling older adults: A cross-sectional analysis. J. Nutr. Sci..

[90] Hengeveld L.M., Wijnhoven H.A.H., Olthof M.R., Brouwer I.A., Harris T.B., Kritchevsky S.B., Newman A.B., Visser M., Health A.B.C.S. (2018). Prospective associations of poor diet quality with long-term incidence of protein-energy malnutrition in community-dwelling older adults: The health, aging, and body composition (health abc) study. Am. J. Clin. Nutr..

[91] Ferguson C.C., Knol L.L., Ellis A.C. (2021). Visceral adiposity index and its association with dietary approaches to stop hypertension (dash) diet scores among older adults: National health and nutrition examination surveys 2011–2014. Clin. Nutr..

[92] Shah R.V., Murthy V.L., Allison M.A., Ding J., Budoff M., Frazier-Wood A.C., Lima J.A., Steffen L., Siscovick D., Tucker K.L. (2016). Diet and adipose tissue distributions: The multi-ethnic study of atherosclerosis. Nutr. Metab. Cardiovasc. Dis..

[93] Ito T., Kawakami R., Tanisawa K., Miyawaki R., Ishii K., Torii S., Suzuki K., Sakamoto S., Muraoka I., Oka K. (2019). *;* et al. Dietary patterns and abdominal obesity in middle-aged and elderly japanese adults: Waseda alumni’s sports, exercise, daily activity, sedentariness and health study (waseda’s health study). Nutrition.

[94] Chan R., Yau F., Yu B., Woo J. (2019). The role of dietary patterns in the contribution of cardiorespiratory fitness in community-dwelling older chinese adults in hong kong. J. Am. Med. Dir. Assoc..

[95] de Breij S., van Hout H.P.J., de Bruin S.R., Schuster N.A., Deeg D.J.H., Huisman M., Hoogendijk E.O. (2021). Predictors of frailty and vitality in older adults aged 75 years and over: Results from the longitudinal aging study amsterdam. Gerontology.

[96] Woo J. (2018). Nutrition and frailty. J. Nutr. Health Aging.

[97] Kojima G., Avgerinou C., Iliffe S., Walters K. (2018). Adherence to mediterranean diet reduces incident frailty risk: Systematic review and meta-analysis. J. Am. Geriatr. Soc..

[98] Rashidi Pour Fard N., Amirabdollahian F., Haghighatdoost F. (2019). Dietary patterns and frailty: A systematic review and meta-analysis. Nutr. Rev..

[99] Silva R., Pizato N., da Mata F., Figueiredo A., Ito M., Pereira M.G. (2018). Mediterranean diet and musculoskeletal-functional outcomes in community-dwelling older people: A systematic review and meta-analysis. J. Nutr. Health Aging.

[100] Chan R., Leung J., Woo J. (2015). Dietary patterns and risk of frailty in chinese community-dwelling older people in hong kong: A prospective cohort study. Nutrients.

[101] Huang C.H., Martins B.A., Okada K., Matsushita E., Uno C., Satake S., Kuzuya M. (2021). A 3-year prospective cohort study of dietary patterns and frailty risk among community-dwelling older adults. Clin. Nutr..

[102] de Haas S.C.M., de Jonge E.A.L., Voortman T., Graaff J.S., Franco O.H., Ikram M.A., Rivadeneira F., Kiefte-de Jong J.C., Schoufour J.D. (2018). Dietary patterns and changes in frailty status: The rotterdam study. Eur. J. Nutr..

[103] Hengeveld L.M., Wijnhoven H.A.H., Olthof M.R., Brouwer I.A., Simonsick E.M., Kritchevsky S.B., Houston D.K., Newman A.B., Visser M. (2019). Prospective associations of diet quality with incident frailty in older adults: The health, aging, and body composition study. J. Am. Geriatr. Soc..

[104] Laclaustra M., Rodriguez-Artalejo F., Guallar-Castillon P., Banegas J.R., Graciani A., Garcia-Esquinas E., Lopez-Garcia E. (2020). The inflammatory potential of diet is related to incident frailty and slow walking in older adults. Clin. Nutr..

[105] Parsons T.J., Papachristou E., Atkins J.L., Papacosta O., Ash S., Lennon L.T., Whincup P.H., Ramsay S.E., Wannamethee S.G. (2019). Physical frailty in older men: Prospective associations with diet quality and patterns. Age Ageing.

[106] Pilleron S., Ajana S., Jutand M.A., Helmer C., Dartigues J.F., Samieri C., Feart C. (2017). Dietary patterns and 12-year risk of frailty: Results from the three-city bordeaux study. J. Am. Med. Dir. Assoc..

[107] Billot M., Calvani R., Urtamo A., Sanchez-Sanchez J.L., Ciccolari-Micaldi C., Chang M.L., Roller-Wirnsberger R., Wirnsberger G., Sinclair A., Vaquero-Pinto N. (2020). Preserving mobility in older adults with physical frailty and sarcopenia: Opportunities, challenges, and recommendations for physical activity interventions. Clin. Interv. Aging.

[108] Huang C.H., Okada K., Matsushita E., Uno C., Satake S., Arakawa Martins B., Kuzuya M. (2021). Dietary patterns and muscle mass, muscle strength, and physical performance in the elderly: A 3-year cohort study. J. Nutr. Health Aging.

[109] Chan R., Leung J., Woo J. (2016). A prospective cohort study to examine the association between dietary patterns and sarcopenia in chinese community-dwelling older people in hong kong. J. Am. Med. Dir. Assoc..

[110] Isanejad M., Sirola J., Mursu J., Rikkonen T., Kroger H., Tuppurainen M., Erkkila A.T. (2018). Association of the baltic sea and mediterranean diets with indices of sarcopenia in elderly women, osptre-fps study. Eur. J. Nutr..

[111] Perala M.M., von Bonsdorff M., Mannisto S., Salonen M.K., Simonen M., Kanerva N., Pohjolainen P., Kajantie E., Rantanen T., Eriksson J.G. (2016). A healthy nordic diet and physical performance in old age: Findings from the longitudinal helsinki birth cohort study. Br. J. Nutr..

[112] Perala M.M., von Bonsdorff M.B., Mannisto S., Salonen M.K., Simonen M., Kanerva N., Rantanen T., Pohjolainen P., Eriksson J.G. (2017). The healthy nordic diet predicts muscle strength 10 years later in old women, but not old men. Age Ageing.

[113] Parsons T.J., Papachristou E., Atkins J.L., Papacosta O., Ash S., Lennon L.T., Whincup P.H., Ramsay S.E., Wannamethee S.G. (2019). Healthier diet quality and dietary patterns are associated with lower risk of mobility limitation in older men. Eur. J. Nutr..

[114] Son B.-K., Akishita M., Yamanaka T., Toyoshima K., Tanaka T., Suthutvoravut U., Iijima K. (2021). Association between inflammatory potential of the diet and sarcopenia/its components in community-dwelling older japanese men. Arch. Gerontol. Geriatr..

[115] Pilleron S., Peres K., Jutand M.A., Helmer C., Dartigues J.F., Samieri C., Feart C. (2018). Dietary patterns and risk of self-reported activity limitation in older adults from the three-city bordeaux study. Br. J. Nutr..

[116] Granic A., Jagger C., Davies K., Adamson A., Kirkwood T., Hill T.R., Siervo M., Mathers J.C., Sayer A.A. (2016). Effect of dietary patterns on muscle strength and physical performance in the very old: Findings from the newcastle 85+ study. PLoS ONE.

[117] Granic A., Mendonca N., Sayer A.A., Hill T.R., Davies K., Siervo M., Mathers J.C., Jagger C. (2020). Effects of dietary patterns and low protein intake on sarcopenia risk in the very old: The newcastle 85+ study. Clin. Nutr..

[118] Bibiloni M.D.M., Julibert A., Argelich E., Aparicio-Ugarriza R., Palacios G., Pons A., Gonzalez-Gross M., Tur J.A. (2017). Western and mediterranean dietary patterns and physical activity and fitness among spanish older adults. Nutrients.

[119] Fu R., Sun Y., Zhai J., Zhang H., Hu Y., Chen H., Cao Y., Qiu Y., Huang X., Kang T. (2021). Dietary patterns and sarcopenia in a chinese population. Asia Pac. J. Clin. Nutr..

[120] Wang X., Ye M., Gu Y., Wu X., Meng G., Bian S., Wu H., Zhang S., Wang Y., Zhang T. (2021). Dietary patterns and sarcopenia in elderly adults: The tclsih study. Br. J. Nutr..

[121] Suthuvoravut U., Takahashi K., Murayama H., Tanaka T., Akishita M., Iijima K. (2020). Association between traditional japanese diet washoku and sarcopenia in community-dwelling older adults: Findings from the kashiwa study. J. Nutr. Health Aging.

[122] Yokoyama Y., Kitamura A., Seino S., Kim H., Obuchi S., Kawai H., Hirano H., Watanabe Y., Motokawa K., Narita M. (2021). Association of nutrient-derived dietary patterns with sarcopenia and its components in community-dwelling older japanese: A cross-sectional study. Nutr. J..

[123] Huang C.H., Okada K., Matsushita E., Uno C., Satake S., Martins B.A., Kuzuya M. (2021). Dietary patterns and intrinsic capacity among community-dwelling older adults: A 3-year prospective cohort study. Eur. J. Nutr..

[124] Gopinath B., Russell J., Kifley A., Flood V.M., Mitchell P. (2016). Adherence to dietary guidelines and successful aging over 10 years. J. Gerontol. A Biol. Sci. Med. Sci..

[125] Assmann K.E., Adjibade M., Andreeva V.A., Hercberg S., Galan P., Kesse-Guyot E. (2018). Association between adherence to the mediterranean diet at midlife and healthy aging in a cohort of french adults. J. Gerontol. A Biol. Sci. Med. Sci..

[126] Samieri C., Sun Q., Townsend M.K., Chiuve S.E., Okereke O.I., Willett W.C., Stampfer M., Grodstein F. (2013). The association between dietary patterns at midlife and health in aging: An observational study. Ann. Intern. Med..

[127] Zhou Y.F., Song X.Y., Wu J., Chen G.C., Neelakantan N., van Dam R.M., Feng L., Yuan J.M., Pan A., Koh W.P. (2021). Association between dietary patterns in midlife and healthy ageing in chinese adults: The singapore chinese health study. J. Am. Med. Dir. Assoc..

[128] Hodge A.M., O’Dea K., English D.R., Giles G.G., Flicker L. (2014). Dietary patterns as predictors of successful ageing. J. Nutr. Health Aging.

[129] Akbaraly T., Sabia S., Hagger-Johnson G., Tabak A.G., Shipley M.J., Jokela M., Brunner E.J., Hamer M., Batty G.D., Singh-Manoux A. (2013). Does overall diet in midlife predict future aging phenotypes? A cohort study. Am. J. Med..

[130] Liguori I., Russo G., Curcio F., Bulli G., Aran L., Della-Morte D., Gargiulo G., Testa G., Cacciatore F., Bonaduce D. (2018). Oxidative stress, aging, and diseases. Clin. Interv. Aging.

[131] Stromsnes K., Correas A.G., Lehmann J., Gambini J., Olaso-Gonzalez G. (2021). Anti-inflammatory properties of diet: Role in healthy aging. Biomedicines.

[132] Roberts S.B., Silver R.E., Das S.K., Fielding R.A., Gilhooly C.H., Jacques P.F., Kelly J.M., Mason J.B., McKeown N.M., Reardon M.A. (2021). Healthy aging-nutrition matters: Start early and screen often. Adv. Nutr..

